# Comparative assessment of total WBC and C-reactive protein counts to procalcitonin levels in subjects having maxillofacial space infection

**DOI:** 10.6026/973206300222476

**Published:** 2026-04-30

**Authors:** Priyadershini A Rangari, Neelam Singh, Alisha Singh, Himani Naresh Singh, Azam Khan, Udaybhan Singh

**Affiliations:** 1 Department of Dentistry, Nandkumar Singh Chouhan Government Medical College, Khandwa, Madhya Pradesh, India; 2 Department of Dentistry, Shri Krishna Medical College Hospital, Muzaffarpur, Bihar, India; 3 Department of Dentistry, ESIC Medical College and Hospital, Indore, Madhya Pradesh, India; 4 Department of ENT, Dr. Laxminarayan Pandey Government Medical College, Ratlam, Madhya Pradesh, India; 5 Department of Oral and Maxillofacial Surgery, Dental College Azamgarh, Uttar Pradesh, India; 6 Department of Oral & Maxillofacial Surgery, R.R. Dental College & Hospital, Udaipur, Rajasthan, India

**Keywords:** Antibiotic regimen, C-reactive protein (CRP), maxillofacial space infections, procalcitonin (PCT), white blood cell (WBC) counts

## Abstract

Orofacial space infections pose life-threatening risks requiring urgent intervention, but trismus/swelling hinders diagnosis. WBC/CRP count help monitors infection. However, PCT (Procalcitonin) offers superior bacterial specificity. Therefore, it is of interest to compare PCT, CRP and WBC levels at admission, 48 h and 96 h in 40 adults with maxillofacial space infections. PCT levels declined significantly earlier (p < 0.05 at admission-to-48h and 48h-to-96h) than CRP/WBC, correlating with clinical improvement and antibiotic response. PCT's rapid normalization outperformed traditional markers in reflecting treatment efficacy amid diagnostic challenges. Thus, we document PCT as a precise biomarker for guiding antibiotic therapy and prognostication in maxillofacial infections.

## Background:

Deep space infections in the cervical areas of the neck have decreased in prevalence over time; however, they remain a concern and the majority of these results from tooth-related or odontogenic causes. They also pose challenges for the maxillofacial surgeon treating these diseases, owing to multiple complications and complex anatomical structures that may be observed despite symptomatic management. Potential mediastinitis, cavernous sinus thrombosis, necrotizing fasciitis, septicemia and airway compromise can develop, followed by infections in the orofacial region and may be fatal [1]. Previous data from the existing literature assessing total count values and procalcitonin levels in subjects with space infections reported reliable and promising results. Procalcitonin is a reliable and potent biomarker that helps in judging bacterial infections with high specificity and sensitivity. Procalcitonin is a precursor derived from the calcitonin hormone and is formed by the C cells present in the thyroid. PCT is also produced by peripheral blood mononuclear cells following bacterial infection and is mediated by interleukin-β (IL-β), tumour necrosis factor α (TNF-α) and IL-6 (interleukin-6) [2].

CRP or C-reactive protein is an acute-phase protein that is synthesized in the liver by hepatocytes and can be assessed in the plasma of a subject when present. The stimulating agent for CRP production is attributed to IL-1 (interleukin-1) release from macrophages in the affected tissues [3]. Owing to the early response of procalcitonin to the bacterial load, it is vital to diagnose and monitor disease progression. In cases of favorable treatment response, PCT values decrease to 50% within 1 day, which helps in assessing antibiotic duration [4]. The majority of studies have assessed lower and upper respiratory tract infections and the literature on potential biomarkers in infections of the maxillofacial region is scarce. Given the promising results of procalcitonin as a biomarker in the existing literature [5]. Therefore, it is of interest to compare total WBC and C-reactive protein counts with procalcitonin levels in subjects with maxillofacial space infection.

## Materials and Methods:

The present observational prospective study was directed at comparing total WBC and C-reactive protein counts with procalcitonin levels in subjects with maxillofacial space infection. The study considered subjects with multiple space infections in the facial region and assessed which could be used as a better measure of treatment response. Reductions in inflammation assessed improvement in clinical symptoms, functional improvement, decreased swelling and the absence of fever. The investigation considered 40 individuals with space infection in the maxillofacial region who presented to the Department of Oral and Maxillofacial Surgery at the Institute during the study period. Verbal informed consent and a written form were attained from all participants. Approval from the institutional Ethical Committee was obtained to conduct the study. The study considered facial space infection subjects aged 20 to 70 years, in whom infection involved more than one orofacial space for clinical assessment and facial swelling was present, with a temperature >37.5°C. Subjects, who had other comorbidities than infections or diabetes, but with facial swelling, were excluded. Demographic data gathered from study subjects included gender, age, glycemic status and vital signs. At the time of presentation, CBC (complete blood counts), PCT and CRP levels were assessed. Ultrasonographic assessment was performed to identify the involved spaces. The admission criteria included neck or facial swelling, no reported cellulitis and one or more of the following: trismus, compromised airway, CRP level > 10 mg/L, WNC counts> 10.8 x 109/L and temperature>37.5°C. Medicine, including 1.2 g IV clavulanic acid, was given by injection. Amoxicillin was given in BD form twice daily and a 500mg intravenous injection of metronidazole was given thrice daily from admission through the day before discharge to manage both anaerobic and aerobic coverage. Foci were removed and the abscess was drained. Analgesics were given for 3 days as an IV injection of paracetamol, 1 gram per dose. Insulin was given according to a sliding scale to achieve glycemic control. Procalcitonin, CRP and CBC were assessed and repeated after 2 days, 4 days and serial readings were recorded. Statistical assessment of the collected data was performed using Student's t-test, with a p-value <0.05 considered statistically significant to evaluate the efficacy of procalcitonin levels, CRP and total counts in identifying treatment response. Comparisons were made over time from admission to 2 days (48 hours) and from 2 days to 4 days (48 to 96 hours) to assess which period better predicted treatment response.

## Results:

In the present research, among 40 subjects assessed, there were 16 males with a mean age of 51.2±9.6 years and diabetes was reported by 18 subjects who were on medication for the same and two had uncontrolled sugar levels (Table 1 - see PDF). The mean glycated hemoglobin (HbA1c) value in study subjects was 6.5, within the range of 5.7 to 7.2. Ludwig's angina was observed in 10 study subjects, with 6 having pterygomandibular and submandibular space involvement and 4 having involvement of the submandibular and buccal spaces. All participants had elevated body temperatures in the range of 37.4 to 38.6°C. Tachycardia was present in all participants, with a mean heart rate of 87 beats per minute. The levels of CRP assessed at the time of admission were in the range of 34-78mg/L and the mean values of CRP at admission, 2 days and 4 days were 57.3±11.35, 46.3±10.57 and 34.53±9.32 mg/L, respectively. The study results showed that mean total counts were in the range of 13.4x106/µL to 19.1 x106/µL, with a mean of 15.42±1.6x106/µL. At 2 days of assessment, the mean total counts were 13.94±2.02 and 12.73±0.6x106/µL, respectively, indicating values higher than normal. Procalcitonin values at admission were 4.54±2.5 ng/ml, with a range of 0.5-9.7 ng/ml. At 2 days, the mean value was 1.057±0.7 ng/ml and at 4 days, it was 0.041±0.02 ng/ml, with the range 0.01-0.05 ng/ml, indicating the values were within the normal limits. There was no variation in the participants' gender or age with respect to PCT values (Table 2 - see PDF). It was observed that, for statistical analysis using the Student's t-test, procalcitonin values decreased from admission to 48 hours and from 48 to 96 hours, with p-values <0.00001 and 0.00001, respectively. Despite a significant decrease in CRP levels and total count over time, the changes were statistically non-significant. This reduction can be closely associated with improvements in clinical parameters across all subjects (Table 3 - see PDF).

## Discussion:

Multiple and severe complications are usually associated with the infections seen in the maxillofacial regions; however, the conventional markers associated with inflammation and infection do not show complete success for the determination of treatment progress and disease severity, as also described by Simon *et al.* in (2008) and Hoeboer *et al.* in (2015) [6, 7]. Clinicians must be able to determine the source of infection and provide appropriate treatment using both surgical and medical interventions. They must evaluate treatment response using both laboratory and clinical parameters [8]. It is vital to avoid prolonged antibiotic use and to discontinue it once satisfactory infection control is achieved [9]. WBCs help produce, transport and distribute antibodies in response to the body's immune system to fight infection. The number of WBCs increases during infection. The rate of change in WBC counts is slow and cannot be considered the sole criterion for assessing disease severity and response to treatment. Levels of CRP in healthy subjects range from 0 to 3 mg/L and increase in proportion to the severity of infection. The levels decreased with the elimination of infection foci; however, levels can remain elevated for weeks [10, 11]. Hence, CRP, along with other markers such as ESR (erythrocyte sedimentation rate) and total counts, can be used as a marker of treatment response with high specificity and sensitivity [12]. A quantitative, descriptive study evaluating WBC count, CRP and PCT serially on day 1 and day 3 in maxillofacial infection patients further confirmed that serial PCT monitoring provides a more objective and dynamic reflection of treatment response compared with WBC and CRP alone, whose levels may remain elevated well beyond clinical resolution, potentially misleading antibiotic discontinuation decisions [13].

Procalcitonin is an excellent biomarker for evaluating infection severity and assessing treatment response. Serum concentration of PCT in normal subjects is <0.1ng/ml. In cases of bacterial infections, values increase in proportion to infection severity and can be assessed within 3-4 hours after infection, immediately after TNF-α and Il-6 release after 90 minutes and 3 hours. Maximum levels are seen at 6-12 hours with a half-life of 24 hours [14]. Procalcitonin converts to calcitonin in C cells of the thyroid gland. In contrast, calcitonin is produced by other sources, such as parenchymal cells, in response to bacterial endotoxins or exotoxins. It does not convert to calcitonin because parenchymal cells lack the CALC1 gene. The majority of studies in critically ill and respiratory tract infection subjects have reported efficacy and safety of antibiotic therapy guided by procalcitonin [15]. A high specificity and sensitivity are observed with procalcitonin compared with WBC or CRP levels, as reported by Simon *et al.* in (2004) [16]. It has also been reported to have high predictive value for determining disease severity and prognosis in pneumonia, summarized by Muller *et al.* in (2010) [17]. A reduction in PCT levels is safely and adequately correlated with the reversal of bacterial infections and allows antibiotic therapy to be discontinued even before completion of the course [18]. PCT levels are also elevated in subjects with malaria, fungal infections, cardiogenic shock and major surgery; however, serial measurement trends show a reduction and clinicians must consider this. Drugs, including alemtuzumab and antilymphocyte globulins, also increase PCT levels 15 these findings correlated with the results of the present study, where PCT levels are increased in subjects with maxillofacial and odontogenic space infections. The improvement in clinical parameters is governed by improved mouth opening, no fever and decreased swelling, which have been correlated with decreases in PCT values, as seen significantly from admission to 2 days and from 2 days to 4 days, with p-values of 0.00001 and <0.00001, respectively. Despite a consecutive reduction in total WBC and CRP levels, these remained towards the higher end of the range, preventing a proper treatment response, which was statistically non-significant. Consistent with the present study, Lalmuanpuia *et al.* (2026) in a prospective cohort of 50 odontogenic infection patients demonstrated that PCT showed the strongest correlation with disease severity (ρ = 0.60), the highest AUC (0.89) in ROC analysis, and the highest OR for complications (3.20), outperforming CRP (AUC = 0.82) and WBC count, thereby reinforcing PCT as the most reliable biomarker for early risk stratification and antibiotic guidance in odontogenic infections [19]. Hence, considering the PCT values, the antibiotic exposure duration can be reduced in accordance with the current consensus on antibiotic therapy.

## Conclusion:

We show that procalcitonin levels are a promising biomarker for assessing treatment response in subjects with space infection in the maxillofacial region and for guiding antibiotic therapy accordingly. Its high sensitivity is evident in its severity compared to other traditionally used markers, such as CRP and total counts, in maxillofacial space infection cases. Proper monitoring can help reduce the duration of antibiotic therapy and minimize antibiotic resistance.
